# Mercury Exposure, Fish Consumption, and Perceived Risk among Pregnant Women in Coastal Florida

**DOI:** 10.3390/ijerph16244903

**Published:** 2019-12-04

**Authors:** Adam M. Schaefer, Matthew Zoffer, Luke Yrastorza, Daniel M. Pearlman, Gregory D. Bossart, Ruel Stoessel, John S. Reif

**Affiliations:** 1 Harbor Branch Oceanographic Institution, Florida Atlantic University, 5600 U.S. 1 N, Ft Pierce, FL 34946, USA; lyrastorza2017@fau.edu (L.Y.); daniel.m.pearlman@gmail.com (D.M.P.); 2 Advent Health, Winter Park, FL 32792, USA; roxyz@comcast.net; 3 Georgia Aquarium, 225 Baker Street NW, Atlanta, GA 30313, USA; gbossart@georgiaaquarium.org; 4 Division of Comparative Pathology, Miller School of Medicine, University of Miami, PO Box 016960 (R-46), Miami, FL 33101, USA; 5 Pre-Birth Centers of America, 8645 N Military Trail, Palm Beach Gardens, FL 33410, USA; rtsmd1@gmail.com; 6 Department of Environmental and Radiological Health Sciences, Colorado State University, 1681 Campus, Fort Collins, CO 80523, USA; john.reif@colostate.edu

**Keywords:** mercury, fish consumption, prenatal contaminant exposure, seafood advisories

## Abstract

Seafood consumption is the primary source of mercury (Hg) exposure, particularly among coastal populations. Hg exposure during pregnancy has been associated with cognitive impairment, as well as decrements in memory, attention, fine motor skills, and other markers of delayed neurodevelopment, although results are conflicting. High Hg hair concentrations in persons from coastal Florida, USA, have been previously reported. The purpose of the current study was to determine the concentrations of total Hg (THg) in the hair of pregnant women from this area and to assess the relationships between THg concentration, knowledge of the risks of mercury exposure, and dietary patterns among participants. Participants (*n* = 229) were recruited at prenatal clinics. Their mean total hair Hg concentration was 0.31 + 0.54 µg/g, lower or similar to US data for women of child-bearing age. Hair THg concentration was associated with consumption of locally caught fish and all seafood, a higher level of education, and first pregnancy. Eighty-five percent of women were aware of the risks of mercury exposure during pregnancy; over half reported a decrease in seafood consumption during pregnancy. Awareness of Hg in fish was marginally associated with lower hair THg concentration (*p* = 0.06) but reduction in seafood consumption during pregnancy was not.

## 1. Introduction

Mercury (Hg) contamination of the marine environment is an important public health concern globally [1]. Inorganic mercury is released into the atmosphere from a variety of industrial activities and subsequently deposited into aquatic and marine ecosystems through rainfall and by dry deposition [2]. Inorganic Hg is converted into methylmercury (MeHg) by sulfate-reducing anerobic bacteria in sediments. MeHg is biomagnified through trophic levels in the marine food web and accumulates in apex predators like dolphins and sharks [3]. Human exposure to mercury occurs primarily through the diet by consumption of seafood, particularly large predatory fish such as swordfish, shark, and albacore tuna [4].

The ability of MeHg to pass through the placenta and cross the blood brain barrier of the fetus resulted in the well-known disaster at Minamata Bay, Japan, where fetal abnormalities, blindness, and severe physical and developmental retardation were reported in the offspring of pregnant women who consumed highly contaminated, locally obtained seafood [5]. The sensitivity of the developing brain to the effects of mercury deposition has been shown in studies of pregnant women exposed through the consumption of seafood, even at relatively low levels of prenatal Hg [6].

Populations living in proximity to marine ecosystems may be at increased risk of exposure to MeHg due to the availability and increased consumption of locally obtained fish and shellfish [7]. In Florida the average adult consumes almost ten times as many grams of seafood per day compared to the general population of the United States [8], potentially increasing the risk of Hg exposure above safe limits. The Indian River Lagoon (IRL), an estuary which extends >250 km and traverses 40% of the eastern coastline in Florida, is an example of a highly impacted environment [7]. Atmospheric deposition of Hg is consistently higher in Florida than in other regions of the country [9]. The increased deposition is compounded by the high capacity for methylation in estuarine waters with low turnover rates and flushing as exists in the IRL [10]. As a result, apex predators like resident bottlenose dolphins (*Tursiops truncatus*) have been reported to have some of the highest concentrations of total mercury (THg) in blood and skin described in the species worldwide [11]. A series of epidemiological studies was conducted to examine THg concentrations in dolphin prey species [12] and dolphins [11].

These studies led us to conduct a study of hair THg concentrations among recreational anglers and coastal residents to assess human exposure and to “Close the Loop” between the wildlife sentinel and human health [7,13]. The THg concentrations in the hair of 135 participants (mean 1.53 ± 1.89 μg/g) was higher than that previously reported for similar populations in the United States [7]. Additionally, individuals who reported consuming locally caught seafood three times a week or more had a threefold risk of having a hair THg concentration above 1.0 µg/g, the US Environmental Protection Agency (EPA) exposure guideline [7,14]. The data raised concern about the risk to the subpopulation most vulnerable to mercury exposure, pregnant women. Therefore, the objective of the current study was to assess mercury concentrations in the hair of pregnant women living in coastal Florida and to determine the relationships between hair THg concentrations, fish consumption, sources of seafood, knowledge of the risks of mercury exposure, and seafood consumption during pregnancy.

## 2. Methods

### 2.1. Study Population

Participants were recruited during prenatal visits to obstetricians at Pre-Birth Centers of the America Center of Excellence (Palm Beach Gardens, Florida and Palm Springs, Florida) and Holmes Medical Center (Ft. Pierce, FL). During the visit, a questionnaire was administered and hair samples collected. Eligibility criteria included (a) being 18 years of age or older; (b) having the ability to understand and consent to participate in the study; (c) being currently pregnant; and (d) being a resident of the Indian River Lagoon region, specifically, Volusia, Brevard, Indian River, St. Lucie, Martin, or Palm Beach counties, all of which border the IRL. Study participants provided informed consent prior to interview. The recruitment process, informed consent, and study protocol were approved by the Institutional Review Board of Florida Atlantic University, IRB #22136-5.

### 2.2. Questionnaire

A standardized and previously validated questionnaire with a 3 month recall period was used to obtain data from participants [7]. Trained interviewers administered the questionnaire in person at pre-natal clinics. A series of questions was asked to obtain demographic information on age, race, education, and residency. A history of previous pregnancies, number of children, and stage of gestation was obtained. Women were asked to recall the overall frequency of seafood consumption using categories of never, once a month or less, once a week, three times a week, or once a day. The sources of the seafood consumed were determined to compare the frequency of obtaining recreationally caught seafood versus that was acquired from commercial sources. Questions were asked to evaluate awareness of the potential effects of Hg exposure on unborn children as well as awareness of the role of fish consumption and specific fish and shellfish species on exposure to mercury. Behavioral changes in patterns of seafood consumption and sources of seafood during pregnancy were assessed, as well as potential changes in smoking and alcohol consumption.

### 2.3. Hair Mercury Analysis

Collection and analysis of hair samples was utilized as a noninvasive method to estimate dietary Hg exposure during the period approximately three months prior to interview [15]. Sterilized stainless steel scissors were used to cut a bundle of hair approximately 3 mm in diameter from the occipital region of each participant which was then immediately placed in a sealed plastic pouch. Approximately 2 cm of the proximal end of each hair sample was sent for laboratory analysis (Quicksilver Scientific, Lafayette, CO, USA. Total mercury concentration was quantified via thermal decomposition, amalgamation, and atomic absorption spectrophotometry according to EPA method 7473, using a Milestone Direct Mercury Analyzer (Milestone Inc., Shelton, CT, USA). Samples were run in triplicate with calibration standards of 100 ppb and 1 ppb with a detection limit of 0.001 µg/g. Total hair mercury concentration was measured and reported as μg/g.

### 2.4. Statistical Analysis

Descriptive statistics (mean, standard deviation, and percentiles) of total hair mercury concentrations were calculated for demographic variables. The THg distribution was assessed for normality using a Kolmogorov–Smirnov (KS) test. THg was log-transformed to meet test assumptions for subsequent statistical analysis. Participant age, weeks of gestation, number of pregnancies, and number of children were categorized for analysis. The relationships between THg and demographic factors, seafood consumption, and seafood sources were examined using an analysis of variance (ANOVA). Potential differences in mean THg concentrations among women who were aware or unaware of the risks of mercury and those who had or had not modified their behavioral practices during pregnancy were compared using a *t*-test.

Variables from the preliminary ANOVAs and *t*-tests with a *p* < 0.20 were initially included in a forward addition multivariate logistic regression model to generate adjusted odds ratios (aOR) and 95% confidence intervals. This model included demographic variables and the a priori hypothesized risk factors of total seafood consumption and awareness that high levels of Hg consumption may be harmful to unborn children. Hair THg was categorized into tertiles (<0.063 µg/g; 0.064–0.229 µg/g; and >0.230 µg/g) for the multivariate model. The lowest tertile of THg was used at the referent category. A second model which retained variables that contributed >10% to the adjusted R^2^ was then run. *p*-values < 0.05 were considered statistically significant. Statistical analysis was conducted using IBM SPSS Statistics version 24 for Windows (IBM Corp. 2011, Armonk, NY, USA).

## 3. Results

### 3.1. Study Population

A total of 244 women were initially recruited between 2015 and 2018. Of those, 229 (93.9%) provided a hair sample and completed the questionnaire. The highest proportion of participants (37.5%) was between the ages of 28 and 32 (Table 1). Caucasians comprised 47.1% of the population, followed by African-American and Latina women. Over half the population had attended college. Twenty-four percent were primigravid while 34.4% had been pregnant four or more times (Table 1).

### 3.2. Hair Mercury Concentrations

Of the 229 women with hair measurements for THg, 69 (30.1%) had concentrations below the detection limit and were assigned an analytical value of 0.50 of the detection limit (0.0005 ppm) for statistical analysis. The mean hair THg concentration of all participants was 0.31ppm (SD = 0.54). Of all women tested, 19 (8.3%) had hair concentrations over 1.0 µg/g, the approximated US EPA reference dose.

Table 1 provides descriptive statistics for demographic variables and pregnancy history as well as mean, standard deviation, median, and percentiles of hair THg concentrations across participant characteristics. The highest concentrations of THg were found in women > 33 years of age. There were significant differences in THg concentrations by race with the highest levels observed among Asian women (mean: 1.18 µg/g). There was a stepwise increase in THg concentrations by level of education with college graduates having the highest concentrations (mean: 0.51 µg/g). Significant differences between THg by county of residence were also reported. A marginally significant (*p* = 0.07) inverse relationship between the number of children and mean hair THg concentration was found with the highest levels in women who had not had children previously. No relationship between duration of pregnancy and hair THg concentration was found.

### 3.3. Seafood Consumption

Seafood consumption once a week or more was reported by 35.5% of respondents, while 17.1% of women did not consume any seafood in the previous three months (Table 2). There was a statistically significant relationship between seafood consumption and hair THg concentration. THg concentrations in hair among those who consumed seafood from the IRL were significantly higher than among women who reported never consuming locally caught items (*p* < 0.01).

### 3.4. Knowledge and Behavior

The majority of participants (85.5%) reported being aware that high levels of Hg may be harmful to the unborn fetus (Table 3). Similarly, 89% of women were aware that some fish can contain high levels of Hg. The mean THg concentrations were slightly higher among those who were not aware of the risks of Hg consumption during pregnancy (mean: 0.41 µg/g) compared to those who were aware (mean: 0.29 µg/g); however, the difference was not statistically significant. Similarly, women who were not aware that some fish contain high levels of Hg had higher (mean: 0.50 µg/g) concentrations than those who were aware of this fact (mean: 0.29 µg/g) but the difference was not statistically significant (*p* = 0.06). Questions assessing knowledge of risks of consuming specific seafood items were also asked. When asked how often one should consume tuna steaks and swordfish, 76.8% of women answered that consumption of these items should be avoided during pregnancy (data not shown). However, only 53.7% of women knew that store-bought swordfish can contain high concentrations of Hg. For species of fish found in the IRL, 54.6% and 47.1%, respectively, knew that locally caught grouper and perch can have high Hg concentrations (data not shown).

Statistically significant differences in THg concentrations were also demonstrated for some behaviors during pregnancy. Women who reported stopping smoking or drinking alcohol after becoming pregnant had lower THg concentrations than those who did not stop (only stopping drinking alcohol was statistically significant). Neither stopping nor reducing seafood consumption during pregnancy was associated with a statistically significant difference in mean hair THg concentration. However, participants who stopped consuming fish and shellfish had a lower, but not statistically significant (*p* = 0.55), THg hair concentration (mean: 0.18 µg/g) compared to those who continued consumption (mean: 0.33 µg/g).

### 3.5. Multivariate Model

Variables considered for inclusion in the final model included age, race, level of education, county of residence, number of children, seafood consumption in the last three months, IRL seafood consumption in the last three months, awareness that seafood can contain mercury, alcohol consumption, and reduction of fish and shellfish consumption. Using a forward stepwise approach, level of education, seafood consumption, and number of children were retained in the final model. The lowest tertile of hair THg (0.00–0.06 µg/g) was used as the referent group for calculation of estimated risks; the highest tertile had >0.23 µg/g (Table 4). Elevated adjusted odds ratios were found for several risk factors. Women with college or post-graduate degrees and those with some college education were, respectively, 11.1 (95% CI = 2.8–44.2) and 4.5 (95% CI = 1.2–16.5) times more likely to have concentrations in the highest tertile of hair THg concentration compared to women with less than a high school education. Primigravid women were 3.9 (95% CI = 1.2–12.9) times more likely to have concentrations in the highest tertile of hair THg compared to women with three or more children. Women who consumed seafood > once a week were 3.2 (95% CI = 1.02–10.19) times more likely to be in the highest tertile of hair THg compared to those who did not consume any seafood. Finally, women who were unaware that high levels of Hg may be harmful to unborn children were 2.8 (95% CI = 0.91–8.4) times as likely to have a hair THg in the highest tertile.

## 4. Discussion

Despite the fact that southern Florida is an area of selective deposition of atmospheric mercury, and that mercury is bioaccumulated in local fish species and apex predators, the mean concentration of THg in this sample of pregnant women (0.31µg/g + 0.54) was similar to or lower than those reported elsewhere in the United States. In the National Health and Nutrition Examination Survey (NHANES), a representative probability sample of the US population, the mean hair Hg concentration among 1726 women of child-bearing age was 0.47 (95% CI = 0.35–0.58) µg/g [16]. Fish consumption during the previous 30 days was directly related to hair Hg concentration. In a population-based cohort study of pregnant women in Massachusetts [17], the mean hair mercury concentration was 0.45 µg/g; a majority of participants reported consuming more than two fish servings weekly, higher seafood consumption than reported in the current study. The concentrations reported here are also lower than those reported for women of childbearing age in other studies from Florida; i.e., 0.37 µg/g [18] and 0.56 µg/g [19]. In our earlier study of coastal Florida residents, the mean hair concentration of THg among women was 0.96 ± 0.74 µg/g [7]. Twenty-five of 62 women (40.3%) had hair THg concentrations > 1.0 µg/g, which corresponds to an EPA reference dose for Hg in hair THg [14]. In that study [7], 56% of participants reported consuming seafood three times a week or more. In contrast, only 8.3% of women sampled in the current study had concentrations above 1.0 µg/g and 16.6% reported eating seafood with that frequency.

Fish consumption patterns and hair mercury concentrations may vary geographically depending on access to local sources of seafood and cultural habits. From an international perspective, the concentrations of THg in hair in this sample of pregnant Florida women were also lower than those reported from countries where seafood consumption is likely to be high. Pregnant women from coastal Italy, Greece, and Taiwan had mean concentrations of 1.06, 136, and 1.73 µg/g in hair, respectively [20,21,22]. Thus, it appears that differences in patterns of seafood consumption are likely to explain differences in mean THg concentrations across populations, even in the same general areas.

Patterns of seafood consumption are also likely to explain higher concentrations of THg in hair among Asian women described above (Table 1). Despite the small sample size, this finding is consistent with other reports on Hg in hair of women of childbearing age in multiple studies conducted in the United States [23,24,25,26]. Fish consumption among Asian women is commonly reported as being higher than among other races in the United States, thus resulting in higher Hg concentrations [10].

Socio-economic status, typically measured as level of achieved education or income, has been reported to be a significant risk factor for Hg exposure among women in previous studies [24,27]. In the current study, level of educational achievement was directly related to hair mercury concentration in a step-wise manner. Women who had completed post-graduate education were 11 times more likely to be in the highest tertile of THg concentration. Level of educational achievement may be related to knowledge of the beneficial effects of fish consumption on cardiovascular health and other endpoints. An annual income of >$75,000 was significantly associated with Hg concentrations in earlier studies in Florida, USA [18,28]. Income is likely related to the affordability of seafood and relatively expensive fish species. Thus, both measures of socio-economic status appear to be related to total seafood consumption and exposure to Hg.

The relationship between seafood consumption and hair mercury concentration has been described in multiple studies. In the current study, the highest concentration of THg in hair was found among women who reported eating seafood three times a week (0.62 µg/g). This concentration was almost four times as high as that found in women who reported consuming no seafood during the previous three months. The consumption of fish and seafood by pregnant women is directly related to the Hg concentrations in the unborn fetus [29]. A dose response relationship between seafood consumption before and during pregnancy, Hg concentration in maternal blood and Hg concentration in cord blood [30], or newborn hair [29], has been previously demonstrated.

We also examined smoking and alcohol use during pregnancy as indicators of health awareness. Participants who continued to smoke and drink alcohol had higher hair THg compared to those who stopped. However, neither behavior remained statistically significant after adjusting for confounding factors in multivariate analyses. These behaviors also did not influence the relationship between seafood consumption and hair Hg concentration in a previous study from Canada [30]. In contrast, in a cohort study in Spain, smoking was associated with THg among pregnant women [31]. It seems plausible that high risk behaviors, such as smoking, consuming alcohol, and consuming foods that pose a health risk during pregnancy, are related, but the relationships are complex and poorly understood.

An important component of this study was an examination of the relationships between knowledge of the risks of seafood consumption and the potential effects of mercury on the fetus, the behavioral practices of this sample of pregnant women, and the concentrations of THg in hair. The majority of participants (85%) reported being aware that Hg may be harmful to the unborn child and 89% were aware that fish can contain high levels of Hg. These data suggest that the educational efforts and advisories circulated by the Florida Department of Health [32] and others are reaching their target audience. However, awareness has not always been effective in reducing hair Hg among women [33]. In the current study, there was no significant difference in the THg in hair between women who reported being aware of the risks of Hg in fish compared to those who were not although the concentrations were lower in the former group. Similarly, although the concentrations among those who stopped consuming seafood during pregnancy (*n* = 19) were nearly half of those who continued, the difference was also not statistically significant. These results are in contrast to previous research supporting the positive impact of dietary intervention among women with elevated hair Hg [33].

It should be noted that the relationships between fish consumption during pregnancy, exposure to mercury, and markers of neuro-development, cognition, fine motor skills, and other neuro-behavioral outcomes are complex and have led to conflicting results in epidemiologic studies [34]. Neurotoxic effects of mercury on the developing brain have been demonstrated in longitudinal studies of population groups with high levels of seafood consumption, such as those in the Faroe Islands where consumption of seafood consisting mainly of pilot whale containing high levels of methylmercury was associated with decrements in verbal skills, memory, and attention at seven years of age [35] and at older ages [36]. Conversely, associations between maternal seafood consumption and childhood cognition were not confirmed in studies of populations in the Seychelles [37,38,39], Great Britain [40], United States [41], and elsewhere [42].

The discrepancies may be explained by the fact that while seafood can be a source of fetal exposure to MeHg, it is also the major source of omega-3 fatty acids which play an essential role in neuro-development. Fish consumption during pregnancy may have a beneficial effect on markers of neuro-development and cognition as shown in several studies [43,44]. Maternal fish consumption had a beneficial effect on language and communication skills in a study of children in Norway but a detrimental effect of MeHg in the highest exposure group was also noted [45]. A threshold effect for maternal hair mercury concentration at 1 µg/g or greater was suggested for inattention, impulsivity, and hyperactive disorders at eight years of age in a cohort study in the USA; however, a protective effect for the same outcomes was associated with maternal consumption of more than two fish meals per week [17]. Therefore, it is critical to balance the risks of mercury exposure with the benefits of seafood consumption during pregnancy and to enhance the understanding of the knowledge, attitudes, and practices of pregnant women concerning this subject.

Several limitations of this study deserve mention. Thirty percent of the samples were below the limit of detection of the assay, thus limiting statistical power. The number of participants in some groups, e.g., women who were unaware of the risks of Hg consumption, as well as the small number of Asian participants, limits the conclusions for these subpopulations. Non-differential misclassification of behaviors was likely to have occurred, likely contributing to the wide confidence intervals for some variables. Questions regarding knowledge of the risks of mercury exposure via seafood consumption were asked at the end of the interview and thus were unlikely to have created response bias. Importantly, we did not assess the level of knowledge regarding beneficial effects of seafood consumption during pregnancy, nor did we assess the proportion of women who took fish oil or other omega-3 supplements during pregnancy and could not assess the effects of these factors on hair mercury concentrations.

## 5. Conclusions

The concentrations of THg in hair of this population of pregnant women from Florida were similar to or lower than those reported in other studies from the United States. Approximately 1 in 10 pregnant women had hair THg concentrations above the approximated EPA reference dose for mercury. Awareness of the risk of prenatal exposure to mercury and the contribution of seafood to mercury exposure was high in this sample of women. From a public health perspective, the results of this study support educating women regarding the risks and benefits of seafood consumption in their early visits for pre-natal care. In view of the serious consequences of prenatal exposure to high concentrations of mercury, continued education on safe sources and species of seafood is warranted. Educational efforts must provide a balanced approach to include information regarding the benefits of fish consumption while minimizing risk by avoiding locally caught seafood or fish species known to contain high levels of mercury.

## Figures and Tables

**Table 1:** Mean concentrations of total mercury in hair (ųg/g) by participant demographics (*n* = 229) and pregnancy history.

Demographic				Percentile			
	*n* (%)	Mean ± SD	Median	75th	90th	95th	*p*-Value **
* Age							0.14
18–22	31 (13.9%)	0.18 ± 0.42	0.08	0.16	0.42	1.49	
23–27	61 (27.4%)	0.28 ± 0.61	0.12	0.26	0.72	1.15	
28–32	66 (29.6%)	0.33 ± 0.50	0.13	0.44	0.92	1.25	
33–44	65 (29.1%)	0.42 ± 0.58	0.17	0.43	4.44	1.67	
**Race**							<0.01
Caucasian	108 (47.2)	0.29 ± 0.51	0.10	0.36	0.92	1.27	
Latina	49 (21.4)	0.24 ± 0.43	0.11	0.27	0.67	1.45	
African-American	61 (26.6)	0.31 ± 0.43	0.12	0.44	1.13	1.4	
Asian/Pacific Islander	7 (3.1)	1.18 ± 1.50	0.62	1.65	-	-	
Other	4 (1.7)	0.17 ± 0.08	0.14	0.25	-	-	
**Level of Education**							<0.01
Less than high school	33 (14.4)	0.15 ± 0.31	0.05	0.15	0.41	1.13	
Graduated from high school	60 (26.3)	0.22 ± 0.39	0.10	0.20	0.72	1.21	
Some college	80 (35.0)	0.32 ± 0.63	0.13	0.33	0.89	1.34	
Graduated from college/post-graduate	55 (24.1)	0.51 ± 0.60	0.30	0.67	1.44	1.89	
**County of Residence**							0.01
Brevard	26 (11.9)	0.38 ± 0.64	0.14	0.49	1.23	2.39	
Indian River	5 (2.3)	0.21 ± 0.24	0.14	0.41	-	-	
St. Lucie	130 (59.9)	0.27 ± 0.45	0.12	0.28	0.90	1.28	
Martin	21 (9.6)	0.16 ± 0.27	0.05	0.28	0.61	1.02	
Palm Beach	20 (9.2)	0.36 ± 1.00	0.36	0.81	1.91	4.25	
Other	15 (6.9)	0.24 ± 0.32	0.11	0.16	0.95	-	
* Number of Pregnancies							0.23
1	55 (24.2)	0.36 ± 0.51	0.14	0.50	1.04	1.89	
2	60 (26.4)	0.39 ± 0.78	0.14	0.34	0.93	2.74	
3	34 (15.0)	0.34 ± 0.51	0.10	0.41	1.37	1.66	
4+	78 (34.4)	0.22 ± 0.30	0.12	0.22	0.62	0.95	
**Number of Children**							0.07
0	73 (32.3)	0.40 ± 0.56	0.15	0.51	1.05	1.90	
1	68 (30.1)	0.38 ± 0.70	0.14	0.36	0.87	1.64	
2	40 (17.7)	0.23 ± 0.39	0.09	0.24	0.92	1.35	
3+	45 (19.9)	0.17 ± 0.27	0.09	0.19	0.41	0.85	
**Number of Weeks Pregnant**							0.80
0–24	77 (33.6)	0.34 ± 0.64	0.16	0.37	0.93	1.18	
25–32	77 (33.6)	0.29 ± 0.48	0.10	0.28	1.21	1.61	
33 or more	75 (32.8)	0.30 ± 0.50	0.13	0.35	0.88	1.36	

* data not provided from all participants; ** *p*-value from ANOVA

**Table 2:** Mean concentrations of total mercury in hair (ųg/g) by frequency of seafood consumption and sources of seafood.

Consumption	*n* (%)	Mean ± SD *	Median	75th	90th	95th	*p*-Value **
**Seafood consumption in the last 3 months**							0.02
Once a day or more	16 (7.0)	0.41 ± 0.74	0.12	0.47	1.62	-	
Three times a week	22 (9.6)	0.62 ± 0.53	0.49	1.18	1.37	1.63	
Once a week	43 (18.9)	0.36 ± 0.73	0.14	0.30	0.96	1.65	
Once a month	108 (47.4)	0.27 ± 0.47	0.11	0.32	0.74	1.23	
Never	39 (17.1)	0.16 ± 0.31	0.01	0.19	0.42	0.94	
**IRL seafood consumption in the last 3 months**							<0.01
> Once a week	14 (6.1)	0.29 ± 0.40	0.90	0.50	1.12	-	
Once a week or less	7 (3.1)	0.53 ± 1.1	0.11	0.36	-	-	
Never	167 (73.2)	0.30 ± 0.53	0.13	0.32	0.86	1.30	
**Fish Sources**							0.81
Half or more caught by you or given to you	13 (5.7)	0.36 ± 0.49	0.14	0.55	1.40	-	
All or most bought from store or restaurant	149 (65.1)	0.40 ± 0.62	0.16	0.44	1.07	1.67	
**Shellfish Sources**							0.27
All or most caught by you or given to you	4 (1.7)	0.45 ± 0.77	0.10	1.24	-	-	
All or most bought from store or restaurant	156 (68.1)	0.35 ± 0.60	0.14	0.36	0.97	1.66	

* Standard deviation; ** *p*-value from ANOVA

**Table 3:** Mean concentrations of total mercury in hair (ųg/g) by knowledge of risks of mercury and practices.

Response	*n* (%)	Mean ± SD *	*p*-Value **
**Aware that high levels of Hg may be harmful to unborn children:**			0.39
Yes	195 (85.5)	0.29 ± 0.48	
No	33 (14.5)	0.41 ± 0.81	
**Aware that some fish can contain high levels of Hg:**			0.06
Yes	203 (89.0)	0.29 ± 0.47	
No	25 (11.0)	0.50 ± 0.92	
**Stopped smoking**			0.21
Yes	46 (59.0)	0.14 ± 0.23	
No	32 (41.0)	0.41 ± 0.63	
**Stopped drinking alcohol**			0.01
Yes	118 (92.9)	0.31 ± 0.47	
No	9 (7.1)	0.66 ± 1.0	
**Reduced fish and shellfish consumption**			0.19
Yes	105 (51.7)	0.38 ± 0.53	
No	98 (48.3)	0.29 ± 0.61	
**Stopped fish and shellfish consumption**			0.55
Yes	19 (9.7)	0.18 ± 0.24	
No	176 (90.3)	0.33 ± 0.56	

* standard deviation; ** *p*-value from independent sample t-test.

**Table 4:** Odds ratios and 95% confidence intervals of being in the higher tertiles of total mercury concentrations in hair (ųg/g), by demographics, knowledge of risks of mercury, and practices.

Tertile THg	00–0.063 µg/g *n* = 76		0.064–0.229 µg/g *n* = 77		>0.230 µg/g *n* = 76	
	aOR	95% CI	aOR	95% CI	aOR	95% CI
**Race**						
Caucasian	1.0	-	Ref.	Ref.	Ref.	Ref.
Latina	1.0	-	2.48	0.98, 6.26	1.50	0.52, 4.27
African-American	1.0	-	1.24	0.50, 3.06	2.03	0.77, 5.36
Asian	1.0	-	-	-	5.54	0.52, 59.15
**Level of Education**						
<High school	1.0	-	Ref.	Ref.	Ref.	Ref.
Graduated from high school	1.0	-	2.77	0.85, 9.07	1.14	0.30, 4.32
Some college	1.0	-	5.75	1.73, 19.13	4.49	1.22, 16.49
Graduated from college/post-graduate	1.0	-	5.64	1.45, 21.83	11.11	2.79, 44.24
**Number of Children**						
0 children	1.0	-	0.70	0.25, 1.91	3.97	1.21, 12.98
1–2 children	1.0	-	0.72	0.30, 1.72	2.29	0.77, 6.84
3+ children	1.0	-	Ref.	Ref.	Ref.	Ref.
**Seafood Consumption in Past 3 Months**						
Never	1.0	-	Ref.	Ref.	Ref.	Ref.
≥Once a week	1.0	-	2.09	0.75, 5.85	3.23	1.02, 10.19
<Once a week	1.0	-	1.82	0.71, 4.65	2.01	0.67, 5.97
**Aware that high levels of Hg may be harmful to unborn children**						
No	1.0	-	1.07	0.35, 3.27	2.76	0.91, 8.41
Yes	1.0	-	Ref.	Ref.	Ref.	Ref.

^a^ Multivariate logistic regression model using the lowest tertile of total hair mercury concentration as the reference group
